# From NAFLD to MASLD and MetALD: Conceptual Shifts in Metabolic Liver Disease and Their Implications for Mexico

**DOI:** 10.3390/medicina62050926

**Published:** 2026-05-09

**Authors:** Alejandro Gutiérrez-Castillo, Ricardo Gutiérrez-Monterrubio, Paul Francisco Domínguez-Cardoso, Andrés Thomson Bejarano-Cayo, Ana Karen Mendoza-Ibáñez, Alondra Rosales-Padron, Sofia Mercedes Narvaez-Chavez, Mario García-Alanís, Luis Fernando Rubio-Acosta, Nahum Mendez-Sánchez, Victor Manuel Paez-Zayas, Ignacio García-Juárez

**Affiliations:** 1 Department of Gastroenterology, Instituto Nacional de Ciencias Médicas y Nutrición Salvador Zubirán, Mexico City 14080, Mexico; alejandro.gutierrezc@incmnsz.mx (A.G.-C.);; 2 Department of Internal Medicine, Instituto Nacional de Ciencias Médicas y Nutrición Salvador Zubirán, Mexico City 14080, Mexico; 3 Department of Gastroenterology, Centro Médico Nacional 20 de Noviembre, Mexico City 03104, Mexico; 4 Department of Neurology and Psychiatry, Instituto Nacional de Ciencias Médicas y Nutrición Salvador Zubirán, Mexico City 14080, Mexico; 5 Liver Research Unit, Medica Sur Clinic & Foundation, Mexico City 14050, Mexico; 6 Faculty of Medicine, National Autonomous University of Mexico, Mexico City 04360, Mexico; 7 Department of Organ Transplantation, Hospital General de Mexico Dr. Eduardo Liceaga, Mexico City 06720, Mexico

**Keywords:** alcohol-associated liver disease, alcohol use disorder, hepatic steatosis, MASLD, MetALD, public health policy

## Abstract

Metabolic dysfunction-associated steatotic liver disease (MASLD), formerly known as non-alcoholic fatty liver disease (NAFLD), is now recognized as the leading cause of chronic liver disease worldwide. The updated terminology reflects a conceptual shift by emphasizing metabolic dysfunction rather than excluding alcohol consumption. This redefinition introduced MetALD, a subtype characterized by the coexistence of cardiometabolic risk factors and moderate alcohol intake. In the world, misclassification of MetALD as MASLD is frequent, often due to underreporting of alcohol consumption, which may distort epidemiological estimates. In Mexico, where both obesity and alcohol use are highly prevalent, this reclassification carries important implications for diagnosis, prognosis, and public health policy. This review summarizes the current evidence on MASLD and MetALD, highlighting their prevalence, diagnostic challenges, and implications for liver transplantation and cancer surveillance. We advocate for integrating these diseases into national non-communicable disease policies and adopting a multidisciplinary, preventive approach tailored to the Mexican context.

## 1. Introduction

Over the last few decades, non-alcoholic fatty liver disease (NAFLD) has been recognized as the hepatic manifestation of metabolic dysfunction; however, its definition—based on the presence of hepatic steatosis in the absence of secondary causes—has been criticized for its conceptual limitations, particularly in populations where metabolic risk factors and alcohol consumption coexist, and there is a lack of pathophysiological specificity [1]. In 2023, an international consensus proposed replacing this terminology with the term metabolic dysfunction-associated steatotic liver disease (MASLD), emphasizing metabolic dysfunction as the main axis of the disease. Nonetheless, this new classification broadens the diagnostic framework by relaxing the exclusion of alcohol intake and expanding the disease spectrum to include metabolic dysfunction-associated steatohepatitis and the metabolic and alcohol-associated liver disease (MetALD), acknowledging the frequent coexistence of metabolic dysfunction and moderate alcohol intake [2].

The transition from NAFLD to MASLD and MetALD represents a paradigm shift in the conceptualization of steatotic liver disease. However, it remains uncertain whether this new classification enhances patient identification, risk stratification or clinical decision-making across specific populations. The question is particularly relevant in countries such as Mexico, which has one of the highest prevalences of obesity, type 2 diabetes, and hazardous alcohol consumption worldwide, alongside structural limitations in access to healthcare, diagnostic infrastructure, and liver transplantation capacity. In these settings, the distinction between MASLD and MetALD may be especially prone to misclassification, potentially affecting epidemiological estimates, risk stratification, and public-health decision-making.

This review aims to critically evaluate the relevance, limitations, and potential need for contextual adaptation of the MASLD/MetALD framework in the Mexican population, integrating epidemiological data, diagnostic challenges, and health system constraints.

### 1.1. Physiopathology MASLD and ALD the Relevance of Dual Injury

The pathogenesis of steatotic liver disease is driven by complex interactions between metabolic dysfunction, insulin resistance, lipotoxicity, and low-grade inflammation. Excess adiposity promotes an increased free fatty acid flux to the liver, the major determinant factor for fatty liver pathogenesis, which ultimately leads to mitochondrial dysfunction, oxidative stress, and hepatocellular injury, finally leading to fibrogenesis. Research into genetic factors underlying these processes has identified several relevant targets, including genes encoding type 1 alpha collagen, sex-hormone binding globulin and patalin-like phospholipase domain-containing protein 3 (PNPLA3), also known as adiponutrin, all of which are associated with an increased risk of disease. In particular, the non-synonymous variant I148M (rs738409 C/G) in the PNPLA3 gene is prevalent in Hispanic and Mexican populations [3]. For example, the frequency of the pathogenic variant is twice as high in Hispanics (0.49) compared with Europeans (0.23) and Africans (0.17) [4]. In a Mexican cohort, the I148M polymorphism was present in 39% of individuals without NAFLD, compared with 50% of patients with NAFLD [3].

On the other hand, alcohol liver disease involves two broad domains. First, genetic and psychiatric factors influence the susceptibility for alcohol dependence. Relevant genetic contributors include variants affecting neurotransmission -such as γ-aminobutyric acid (GABA) pathways- as well as genes involved in alcohol metabolism, including alcoholic dehydrogenase and acetaldehyde dehydrogenase enzymes [5]. Second, a distinct group of genes modulates the hepatic response to alcohol consumption, thereby influencing disease progression. These mechanisms include increased production of inflammatory mediators, oxidative stress, acetaldehyde toxicity, gut microbiota dysbiosis, and enhanced intestinal permeability, which together promote endotoxemia and hepatic inflammation. Notably, the A1 polymorphism of the dopamine receptor D2 (DRD2) gene has been associated with a higher threshold for dopaminergic stimulation, leading to increased alcohol intake and greater risk of abuse. This allelic variant shows a particularly high prevalence among Amerindian population, including Pima (83%) and Mayan (71%) groups in Mexico [6].

In this context, MetALD emerges as a particularly relevant clinical entity in the Mexican population, where metabolic dysfunction and hazardous alcohol consumption frequently coexist and interact with a distinct genetic background. The high prevalence of obesity, type 2 diabetes, and alcohol use—combined with the increased frequency of risk variants such as PNPLA3 I148M—creates a synergistic environment that promotes both hepatic steatosis and accelerated disease progression [5,6]. These overlapping mechanisms not only support the biological plausibility of MetALD but also suggest that its burden may be disproportionately high in this population.

### 1.2. Epidemiology of MASLD: A Growing Burden

In Mexico, liver-related diseases are the sixth leading cause of death among the economically active population [7]. The etiologic profile of cirrhosis has shifted substantially over time. In 2002, the main causes were alcohol (39.5%), Hepatitis C virus (HCV) infection, (36.6%) and cryptogenic cirrhosis (10.4%) [8]. Between 2012 and 2017, HCV (36.2%) became the leading etiology, followed by alcohol (31.2%) and MASLD (23.2%) [9]. More recent studies have identified MASLD (30–42.8%) as the most frequent cause of cirrhosis, followed by alcohol (23.8–24%) and HCV (12.2–23%) [10,11]. It is projected that by 2050, MASLD and alcohol will together account for up to 90% of chronic liver disease cases in Mexico [12].

The growing prevalence of MASLD is undeniable, as it has emerged as the leading cause of cirrhosis worldwide, with an estimated prevalence of 30% in the adult population [13]. In Latin America, the situation is even worse, given the high rates of obesity, diabetes, sedentary lifestyles and hypercaloric diets [10]. Mexico mirrors this trend: according to the latest National Health and Nutrition Survey (ENSANUT 2024), more than 70% of the Mexican adults are overweight or obese [14], and the prevalence of steatotic liver disease (SLD) in these populations may exceed 50% [15].

Although the increasing prevalence of MASLD in Mexico parallels global trends, the available epidemiological data present important limitations. Most estimates derive from hospital-based or urban cohorts, which may overrepresent advanced disease and limit generalizability to the broader population. Furthermore, the frequent underreporting of alcohol intake may lead to misclassification between MASLD and MetALD, potentially distorting national prevalence estimates. Therefore, while MASLD appears to be emerging as the leading cause of cirrhosis in Mexico, the true distribution of steatotic liver disease subtypes remains uncertain, and current estimates may underestimate the burden of alcohol-related disease.

### 1.3. Clinical Impact and Prognosis of MASLD

**Figure 1:**
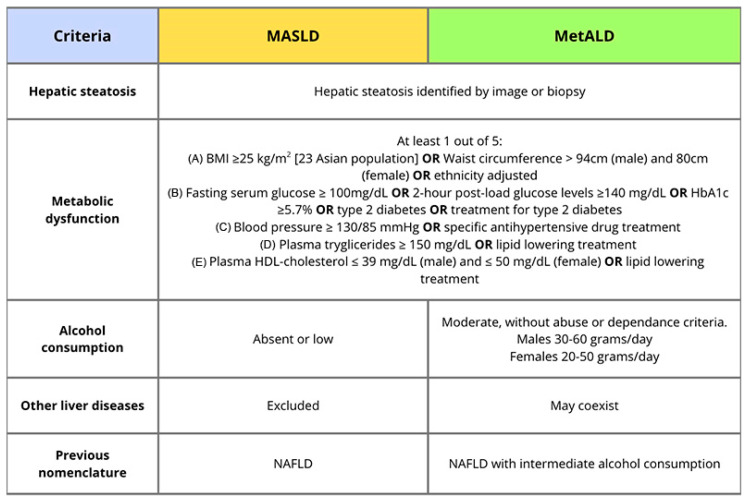
Definition of Metabolic Dysfunction-Associated Steatotic Liver Disease (MASLD) and Metabolic and Alcohol-associated Liver Disease (MetALD) according to 2024 EASL-EASD-EASO Clinical Practice Guidelines on the management of MASLD.

MASLD, defined as the presence of hepatic steatosis in conjunction with at least one cardiovascular risk factor [2] (Figure 1), exhibits a highly variable clinical course. Although the classical paradigm of chronic liver disease follows a linear progression from inflammation and fibrosis to cirrhosis and an increased risk of hepatocellular carcinoma (HCC), MASLD presents several distinctive features.

First, it is often difficult to suspect, as approximately 22–33% of patients would have advanced fibrosis with normal ALT levels (<40 U/L) [16] and around 7–20% of the population classified as having MASLD are non-obese [17], defined by a body mass index lower than 25 kg/m^2^. Second, it is not a disease easy to diagnose, as the hepatic venous pressure gradient—the gold standard for diagnosing portal hypertension—may remain below 10 mmHg despite the clinical manifestations of portal hypertension, mainly due to a presinusoidal component [18,19]. Finally, MASLD is one of the few risk factors of HCC in the absence of cirrhosis. Although the annual incidence of HCC in non-cirrhotic MASLD is estimated at <0.2%, approximately one-quarter to one-third of MASLD-related HCC occur without cirrhosis. The latter represents a major clinical challenge, as current guidelines do not recommend routine surveillance in non-cirrhotic MASLD, potentially leading to delayed diagnosis [20].

### 1.4. The Alcohol Burden and the Necessity of an Intermediate Category

Alcohol consumption remains a global challenge. According to WHO in 2018 around 2.3 billion people worldwide were current drinkers, and by 2019, 2.6 million deaths worldwide were attributable to alcohol consumption [21,22]. For example, in the US by 2022, 177 million people reported that they drank in the past year and 61.2 million people aged 12 and older had at least one binge episode in the preceding month; and by 2020–2021 the average number of deaths attributed to excessive alcohol use increased to 178,307 [23].

In Mexico, the most recent national survey on alcohol use revealed a notable increase in harmful drinking patterns: monthly binge drinking rose from 13.9% to 19.8%, and daily alcohol use from 0.8% to 2.9% among adults aged 18–65 years. Among adolescents, lifetime alcohol use reached 39.8%, monthly consumption 16.1%, and binge drinking episodes increased from 4.3% to 8.3% over the same period. These findings indicate persistently high per capita alcohol consumption (≈7–10 L/year per adult) and escalating hazardous drinking behaviors, particularly among younger populations, underscoring the urgent need for targeted public health interventions [24]. In a prospective study in Mexico City that recruited 150,000 adults from 1998 and 2004, there were 3067 deaths from alcohol-related causes, and, compared with occasional light drinkers, occasional and regular heavy episodic drinking was associated with 20% and 80% higher alcohol-related mortality, respectively [25].

In Mexico, alcohol consumption has deep historical and cultural roots. The emergence of sedentary societies was accompanied by the discovery and consumption of alcoholic beverages. One of the first beverages of record were the ones made by the endemic agave plant. Today, Mexicans rank among the countries with the highest alcohol consumption in the region and report a high mortality rate from alcohol-related liver disease [6].

To characterize a disease entity defined by the synergistic effect of metabolic dysfunction and alcohol consumption, associated with worse clinical outcomes, a new category was needed [26]. MetALD is defined by the presence of hepatic steatosis with at least one cardiometabolic risk factor and moderate alcohol intake, specified as 140–350 g/week (20–50 g/day) for women and 210–420 g/week (30–60 g/day) for men [26,27]. However, the diagnostic threshold defining “moderate” alcohol intake remains arbitrary and may not reflect biological susceptibility or regional drinking patterns. Also, the burden of alcohol-related disease may be underestimated, as alcohol use remains stigmatized in many cultures.

Alcohol intake has been shown to be underreported in 10–55% of patients diagnosed with MASLD [28]. In the MAESTRO trial, for example, up to 58% of participants underestimated their alcohol consumption [29]. The advent of indirect alcohol biomarkers, such as phosphatidylethanol (PEth) and ethyl glucuronide (EtG), has demonstrated that approximately 17–29% of patients previously classified as MASLD may in fact be reclassified as MetALD [29,30]. Among these, PEth appears particularly promising as an objective biomarker. It can detect ethanol exposure up to 12 days after a single binge episode, remains stable in whole blood for up to 28 days at room temperature, and is not significantly affected by age, race, or ethnicity. A cutoff value of 20 ng/mL indicates elevated alcohol consumption in the general population, while a threshold of 35 ng/mL differentiates MASLD from MetALD with ≥90% specificity [31]. Unfortunately, the implementation of PEth testing in Mexico remains a challenge. Although PEth/EtG studies suggest substantial reclassification rates in other populations, it remains unclear whether these estimates apply to Mexico, where drinking patterns, stigma and healthcare access differ. And, since both biomarkers are unavailable in Mexico, there is currently no ideal, accessible tool to objectively assess alcohol consumption in Mexico.

These emerging data may significantly reshape our understanding of MASLD epidemiology. Current estimates suggest that within the spectrum of SLD, MASLD accounts for approximately 91% of cases, followed by MetALD (6%) and alcohol-related liver disease (ALD) (3%). However, in simulated scenarios accounting for underreported alcohol consumption, the proportion of MetALD among patients previously classified as MASLD could range from 9% to as high as 50% [32].

Consequently, in populations with high rates of both metabolic dysfunction and alcohol consumption, such as Mexico, MetALD may be misclassified as MASLD when alcohol intake is unrecognized. Moreover, its diagnostic criteria may not be universally applicable across populations with different patterns of alcohol consumption and genetic susceptibility. These limitations carry important clinical implications, as inaccurate classification may affect prognosis assessment, eligibility for surveillance strategies, and therapeutic decision-making.

### 1.5. The Clinical Consequences of This New Category

Although MASLD is the most prevalent subtype (31–38%), MetALD and ALD account for the majority of liver-related hospitalizations, transplants, and deaths. These patients also show higher rates of HCC, reduced kidney function, and hyperuricemia [29]. In Germany, for instance, alcoholic cirrhosis represents more than 50% of cirrhosis-related hospitalizations, whereas MASLD accounts for only 3%. Combined liver-related mortality for MetALD and ALD reaches 45%, compared with just 4% in biopsied MASLD cases [33].

Cause-specific mortality also differs across the SLD spectrum. Although cardiovascular disease remains the leading cause of death in both non-cirrhotic and cirrhotic MASLD, MetALD, and ALD, patients with cirrhotic MetALD or ALD show a higher proportion of liver-related mortality compared with those with cirrhotic MASLD, in whom cardiovascular disease continues to predominate [32]. Table 1 shows differences between MASLD and MetALD, and their implications for Mexico.

### 1.6. Impact of the Emerging MASLD/MetALD Spectrum on Liver Transplantation Capacity

The growing burden of MASLD and MetALD is reshaping the epidemiology of advanced liver disease and liver transplantation (LT). As viral hepatitis declines due to effective antiviral therapies, metabolic dysfunction and alcohol-related liver disease have emerged as leading indications for LT [8,17]. However, the clinical and systemic implications of this epidemiological shift vary substantially across regions.

From a clinical perspective, patients with MASLD and MetALD present distinct challenges. These individuals frequently exhibit advanced age, obesity, type 2 diabetes, and cardiovascular comorbidities, all of which are associated with increased perioperative risk and post-transplant complications. In the context of MetALD, the coexistence of alcohol use introduces an additional layer of complexity, necessitating structured psychosocial assessment, the evaluation of relapse risk (17–30%), and longitudinal monitoring [34]. Collectively, these factors may delay listing or limit transplant eligibility. Emerging evidence suggests that the combination of metabolic dysfunction and alcohol exposure may accelerate disease progression and reduce the likelihood of clinical stabilization prior to transplantation [35]. This has important implications for transplant timing and prioritization, particularly in settings where access to transplantation is already constrained.

In Mexico, the impact of MASLD and MetALD on LT must be understood within the context of a markedly constrained transplant system. National data show a widening gap between liver disease burden and transplant capacity: liver-related deaths increased from 31,528 in 2008 to 40,052 in 2023, whereas LT activity rose only modestly from 97 to 297 procedures annually (0.87 to 2.5 per million population). As a result, in 2023, transplantation was available to only 0.7% of patients who require it. This mismatch reflects multiple structural barriers, including low waitlist registration rates, limited donor availability, and systemic inefficiencies. Access to LT is further restricted by marked geographic centralization—over 97% of transplants are performed in three states—and by fragmentation of the healthcare system, which further complicates referral and delays evaluation. These limitations are compounded by insufficient funding, lack of reimbursement mechanisms, and shortage of specialized personal, all of which constrain the scalability of transplant services. In this context, the rising prevalence of MASLD and MetALD is likely to increase the demand for transplantation within an already capacity-limited system, potentially exacerbating existing inequities in to specialized care [36,37,38,39].

The accurate classification of steatotic liver disease subtypes is therefore critical in this context. Misclassification of MetALD as MASLD—driven by underreporting of alcohol consumption—may lead to underestimation of disease severity, inappropriate risk stratification, and suboptimal allocation of resources [37]. This issue is particularly relevant in Mexico, where objective assessment of alcohol intake is not routinely implemented.

Taken together, these observations suggest that the MASLD/MetALD spectrum not only increases the demand for liver transplantation but also introduces additional layers of clinical and operational complexity. Addressing these challenges will require integrated strategies focusing on early disease detection, standardized assessment of alcohol use, and improved coordination between metabolic, hepatology, and addiction care services. In resource-limited settings such as Mexico, strengthening these pathways may be essential to prevent further strain on an already constrained transplant system.

### 1.7. MASLD as a Global Public Health Challenge: From Conceptual Recognition to Implementation

The incorporation of MASLD into non-communicable disease (NCD) frameworks highlights its growing recognition as a systemic cardiometabolic condition rather than an isolated liver disorder. NCDs—including cardiovascular disease, type 2 diabetes, chronic respiratory diseases, and cancer—account for approximately 74% of global mortality, highlighting the scale at which metabolic disorders contribute to population health burden [40]. Within this context, MASLD has emerged as a key component of this spectrum, given its close association with cardiometabolic risk factors and its contribution to both hepatic and extrahepatic outcomes [41,42].

In response, international initiatives have sought to position MASLD as a public health priority through the development of comprehensive, multidisciplinary strategies and its integration into national NCD agendas [43]. These efforts emphasize prevention, early detection, and coordinated care models, supported by the implementation of non-invasive diagnostic tools and population-based interventions [44].

However, the transition from conceptual recognition to effective implementation remains uneven. Many proposed strategies assume the availability of structured healthcare systems, access to diagnostic resources, and integrated care pathways. In practice, the feasibility of these approaches depends on local healthcare infrastructure, resource allocation, and system-level organization, which vary considerably across regions [44,45].

Within this context, the integration of MASLD into public health strategies should be understood not merely as inclusion within NCD frameworks, but as a process requiring adaptation to local conditions. Prevention-oriented approaches—such as lifestyle interventions, early risk stratification, and the incorporation of liver assessment into routine care—are inherently dependent on scalability, coordination, and sustainability within existing healthcare systems.

The emerging concept of “liver health” offers a unifying framework by linking hepatic outcomes with broader metabolic health; however, its implementation requires alignment with healthcare delivery models and resource availability across different settings [46]. Ultimately, the impact of MASLD as a public health priority will depend less on its conceptual redefinition and more on the capacity to translate global recommendations into context-sensitive, scalable strategies.

The expanding burden of MASLD and MetALD therefore extends beyond clinical hepatology and calls for coordinated policy responses. Recent global initiatives have emphasized the need for integrated care pathways, early detection strategies, and regulatory alignment to reduce long-term societal and economic costs [47]. These recommendations are particularly relevant for middle-income settings, where high cardiometabolic risk prevalence intersects with healthcare fragmentation and limited access to specialized care. In such settings, prevention, early fibrosis detection, and structured alcohol risk assessment may represent the most cost-effective and equitable strategies to mitigate future demand for advanced liver disease care and liver transplantation. Aligning national liver health strategies with global NCD agendas may therefore be critical to preventing the MASLD/MetALD spectrum from further amplifying disparities in access to specialized liver care.

## 2. Conclusions

The transition from NAFLD to MASLD and MetALD represents a meaningful conceptual advance toward more pathophysiologically-oriented classification of steatotic liver disease. However, its clinical value is not inherently universal. In Mexico, the high coexistence of metabolic dysfunction and alcohol consumption, combined with the underreporting of alcohol intake and limited diagnostic resources, creates a substantial risk of misclassification. This limitation may compromise epidemiological accuracy, clinical decision-making, and healthcare planning.

Currently, there is limited evidence demonstrating that the MASLD/MetALD framework improves patient identification or clinical outcomes in the Mexican population. Moreover, the absence of validation studies and national consensus further limits its immediate applicability. Rather than direct implementation, a context-sensitive adaptation is warranted. This should include improved assessment of alcohol intake, broader access to diagnostic tools, and formal evaluation of the classification system in local populations.

Ultimately, the success of the MASLD/MetALD framework will depend not on its conceptual clarity, but on its ability to improve real-world clinical outcomes. Without careful adaptation, there is a risk that this reclassification may add complexity without delivering meaningful benefit in settings where the burden of disease is greatest. Future research should prioritize region-specific validation to ensure that this evolving framework translates into meaningful clinical and public health benefits.

## Figures and Tables

**Table 1:** Conceptual, clinical, and implementation differences between MASLD and MetALD, with specific considerations for the Mexican context.

Domain	MASLD	MetALD	Implications for Mexico
**Definition**	Hepatic steatosis + ≥1 cardiometabolic risk factor, minimal alcohol intake	Hepatic steatosis + cardiometabolic risk factors + moderate alcohol intake	High overlap between metabolic dysfunction and alcohol consumption may blur diagnostic boundaries
**Pathophysiology**	Primarily driven by insulin resistance, lipotoxicity, and chronic inflammation	Combined metabolic dysfunction and alcohol-induced oxidative stress and gut–liver axis disruption	Synergistic mechanisms may accelerate disease progression in populations with dual exposure
**Epidemiology**	Most prevalent SLD subtype globally (~30%)	Lower reported prevalence (~5–10%), likely underestimated	True prevalence uncertain due to underreporting of alcohol consumption and lack of population-based studies
**Diagnostic challenges**	Often asymptomatic; normal ALT possible; limited access to non-invasive tools	Requires accurate quantification of alcohol intake; biomarkers not widely available	Underreporting of alcohol use and limited access to PEth/EtG testing increase misclassification risk
**Misclassification risk**	May include patients with unreported alcohol intake	Frequently underdiagnosed due to stigma and reporting bias	High—estimated reclassification rates may significantly alter epidemiological data
**Clinical outcomes**	Variable progression; cardiovascular mortality predominant	Higher rates of liver-related complications, HCC, and mortality	Potential underestimation of disease severity if MetALD is misclassified as MASLD
**HCC risk**	Can occur without cirrhosis; low annual incidence	Higher liver-related mortality and complication rates	Challenges for surveillance strategies in resource-limited settings
**Transplant implications**	Increasing indication due to metabolic burden	More complex candidate selection due to alcohol use	Limited transplant capacity may be disproportionately affected by MetALD burden
**Health system impact**	Requires integration into NCD frameworks	Requires combined metabolic and addiction management	Healthcare fragmentation and limited resources complicate implementation
**Evidence gaps**	Limited data in middle-income countries	Even fewer validation studies	Lack of national validation studies represents a critical gap
**Need for adaptation**	Developed from global consensus	Emerging category with evolving criteria	Context-specific adaptations likely needed for accurate implementation in Mexico

## Data Availability

No new data were created or analyzed in this study. Data sharing is not applicable to this article.

## Abbreviations

The following abbreviations are used in this manuscript:

ALD	Alcohol-related liver disease
HCC	Hepatocellular carcinoma
MASLD	Metabolic dysfunction-associated steatotic liver disease
MetALD	Metabolic and alcohol-associated liver disease
NAFLD	Non-alcoholic fatty liver disease
Peth	Phosphatidylethanol
SLD	Steatotic liver disease
